# Mapping of elasticity and damping in an α + β titanium alloy through atomic force acoustic microscopy

**DOI:** 10.3762/bjnano.6.79

**Published:** 2015-03-18

**Authors:** M Kalyan Phani, Anish Kumar, T Jayakumar, Walter Arnold, Konrad Samwer

**Affiliations:** 1 Metallurgy and Materials Group, Indira Gandhi Centre for Atomic Research, Kalpakkam-603102, Tamil Nadu, India; 2 Department of Materials and Materials Technology, Saarland University, Campus D 2.2, D-66123 Saarbrücken, Germany; 3 1. Physikalisches Institut, Georg-August-Universität, Friedrich Hund Platz 1, D-37077 Göttingen, Germany

**Keywords:** atomic force acoustic microscopy, contact resonances, damping, indentation modulus, Ti-6Al-4V

## Abstract

The distribution of elastic stiffness and damping of individual phases in an α + β titanium alloy (Ti-6Al-4V) measured by using atomic force acoustic microscopy (AFAM) is reported in the present study. The real and imaginary parts of the contact stiffness k^*^ are obtained from the contact-resonance spectra and by using these two quantities, the maps of local elastic stiffness and the damping factor are derived. The evaluation of the data is based on the mass distribution of the cantilever with damped flexural modes. The cantilever dynamics model considering damping, which was proposed recently, has been used for mapping of indentation modulus and damping of different phases in a metallic structural material. The study indicated that in a Ti-6Al-4V alloy the metastable β phase has the minimum modulus and the maximum damping followed by α′- and α-phases. Volume fractions of the individual phases were determined by using a commercial material property evaluation software and were validated by using X-ray diffraction (XRD) and electron back-scatter diffraction (EBSD) studies on one of the heat-treated samples. The volume fractions of the phases and the modulus measured through AFAM are used to derive average modulus of the bulk sample which is correlated with the bulk elastic properties obtained by ultrasonic velocity measurements. The average modulus of the specimens estimated by AFAM technique is found to be within 5% of that obtained by ultrasonic velocity measurements. The effect of heat treatments on the ultrasonic attenuation in the bulk sample could also be understood based on the damping measurements on individual phases using AFAM.

## Introduction

The physical and mechanical properties of the individual phases govern the respective properties of the multiphase structural materials. The knowledge of elastic properties of the individual phases is important for studying their deformation behavior, crack nucleation and propagation, dislocation activity and interaction with grain boundaries and also even helps in understanding the bulk elastic properties of multiphase materials [1–2]. Over the last two decades many contact-resonance-based atomic force microscopy (AFM) techniques, such as ultrasonic atomic force microscopy (UAFM) [3], and atomic force acoustic microscopy [4] have emerged for the characterization of elastic properties of materials with nanometer resolution. UAFM and AFAM work with a similar principle and only vary in the excitation of the cantilever. In UAFM the cantilever is excited by attaching a transducer to the cantilever base. In AFAM, the transducer is placed under the sample and periodic displacements of the sample are sensed by the cantilever when in contact. Rabe et al. [4], Hurley et al. [5] have discussed in detail the development of contact-resonance force microscopy techniques for quantitative measurements of nanomechanical properties. Ogi et al. [6] have studied elastic and damping properties in a dual-phase steel by using resonance ultrasound microscopy (RUM), which is a contact-resonance based technique but limited to micrometer resolution. An improved UAFM technique was used for mapping the resonance frequency and the quality factor, Q, in carbon reinforced plastics composites [7]. In recent years, AFAM has been extensively used to determine elastic stiffness or damping properties in nano-crystalline nickel [2], PMMA films [8], NiMnGa films [9], Arabidopsis plant [10], polystyrene–propylene blends [11], nickel base alloys [12–13], ferritic steels [13], and metallic glasses [14]. Besides contact-resonance based methods, multi-frequency AFM techniques have also been used for measurement of elastic and damping properties in living cells [15], proteins [16] and polymers [17–18]. They have been found to be useful in probing material properties with enhanced sensitivity, less surface damage and also at larger distances [18–19]. However, these techniques were developed for soft materials with moduli smaller than 10 GPa.

The type and amount of various phases formed during thermal/thermo-mechanical treatments influence both the mechanical as well as the elastic properties of α + β titanium alloys [1]. By varying the volume fractions of the two phases present in the alloy, various combinations of strength and toughness can be achieved. Ti-6Al-4V combines the benefits of high strength, light weight, formability and corrosion resistance and finds applications in aircraft structural components, aerospace fasteners, high-performance automotive parts, marine applications, medical devices, and sports equipment. Owing to the good corrosion resistance and biocompatibility, Ti-6Al-4V is also widely used in making load-bearing metal implants [20]. Different studies have been reported on phase transformations [21] and mechanical property variations with various phases [22] in Ti-6Al-4V. Kumar et al. [1] have successfully mapped the indentation modulus of α- and β-phases in a Ti-6Al-4V alloy by using AFAM while using a cantilever dynamic model in which damping, however, was neglected. In this paper, we report mapping of elastic modulus and damping using a modified cantilever dynamic model in various phases, such as α, β and α′ in Ti-6Al-4V alloy heat-treated at different temperatures. The modulus of individual phases measured by AFAM has been used to calculate the average modulus of the alloy in different heat treatment conditions, which is correlated with the modulus obtained by the bulk ultrasonic velocity measurements.

## Measurement of elastic stiffness and damping through AFAM

**Figure 1:**
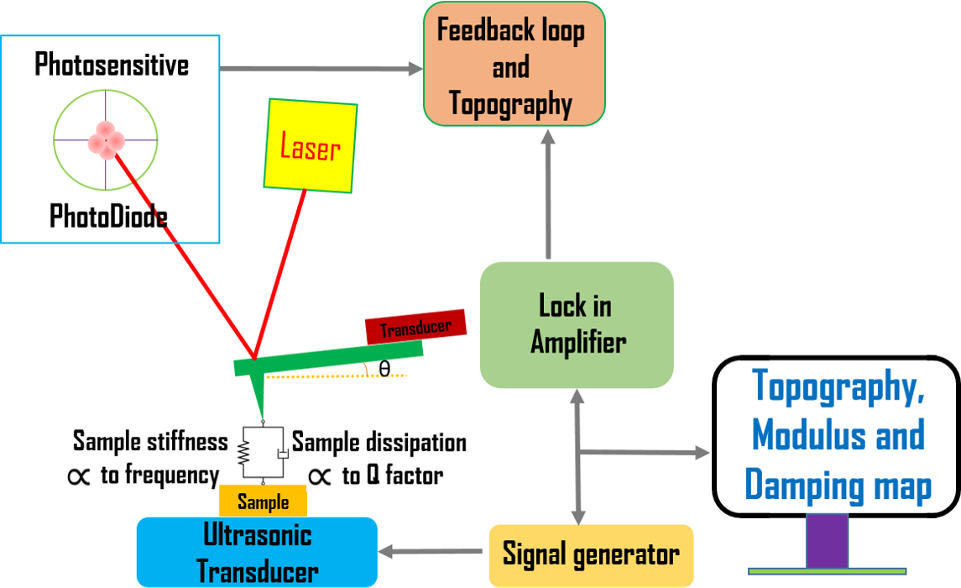
Schematic setup of atomic force acoustic microscopy.

In AFAM, longitudinal waves are injected into the specimen from its bottom by using a transducer, which leads to periodic displacements of the specimen surface. These vibrations are sensed by a probe (cantilever + tip) during contact. The amplitude of the vibrations of the probe after excitation at various frequencies is detected through the AFM photodiode signal by the use of a lock-in-amplifier [14]. The response of the probe as a function of the frequency can be acquired by sweeping a wide range of frequencies. The obtained contact-resonance spectra are used to calculate the contact stiffness k* and the local contact damping *E*″/*E*′ by employing suitable models for the tip–specimen contact, and in turn enabling one to image and to measure the local elasticity or the storage modulus *E*′ and the damping or loss modulus *E*″ of the specimen surfaces with a spatial resolution of a few tens of nanometers. A schematic representation of set-up of AFAM is shown in Figure 1.

Detailed descriptions of the theory and calculations of the indentation modulus and damping are given elsewhere [4,23–24]. In particular for measuring contact damping, the cantilever model must be taken into account [8], and this has been applied recently to metallic glasses by Wagner et al. [24]. They have successfully demonstrated a quantitative approach to determine the local internal friction or loss at a nanometer scale, using the evaluation procedure of the cantilevers distributed mass model with damped flexural modes on amorphous PdCuSi metallic glass.

The solution of the characteristic dispersion equation of the ‘cantilever dynamics’ model was presented in many publications, also in [24]:

[1]
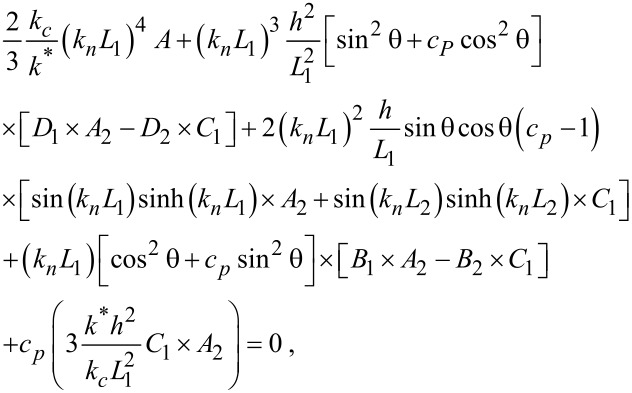


where


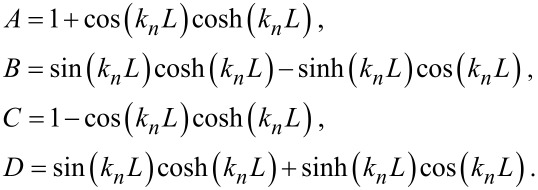


The subscripts 1, 2 used here are for *L*_1_ and *L*_2_ and hold for all *A, B, C and D* for example:


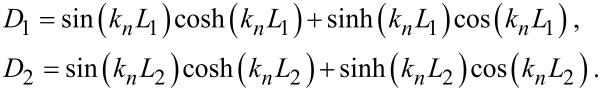


*L* is the total cantilever length and *L*_1_ is the distance between the tip position and the free end of the cantilever, and *L*_2_ = *L* − *L*_1_*.* The values used for the calculations in the present study are *L*_1_/*L* = 0.94, the angle of inclination of the cantilever θ = 12° and the ratio of the lateral to the vertical contact stiffnesses *c_p_* = 0.85 given by the Poisson’s ratio of the materials examined [4,23].

When damping is taken into account, the wave-vector *k_n_* describing the motion of the cantilever becomes complex with *a_n_* being its real part and *b_n_* being its imaginary part. The imaginary part is caused by the damping of the tip–specimen configuration, i.e., the damping in the contact zone, and the damping of the cantilever motion in air. The cantilever resonance frequencies determine the real part *a_n_*, and the imaginary part *b_n_* is determined by the width of the contact resonance curves [8,11]:

[2]
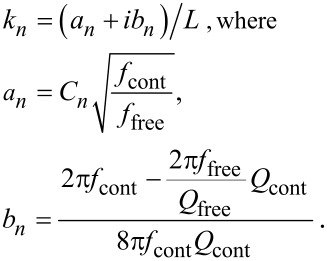


Here, *n* = 1, 2,…∞ are the mode numbers, e.g., *C*_1_ = 1.875 for the first free resonance. In Equation 2 it is neglected that the damping of the cantilever during its motion in air is not the same for the free cantilever and the cantilever in contact [24]. However, due to the relative high contact damping *E″*/*E′*, which we observe in our experiments, we neglect this effect.

The values for *a_n_* and *b_n_* are obtained by fitting Lorentzians to the experimentally obtained resonances curves of the free and contact resonances. Due to the local damping in the contact zone the contact stiffness becomes a complex quantity, i.e., k^*^ = *k_r_* *+ ik_i_*, where *k_r_* is the real part of the contact stiffness and *k_i_* is the imaginary part of the contact stiffness. In case there is viscous damping in the contact zone, *k_i_ =* ωγ, where ω is the angular frequency. Neither *k_r_* nor *k_i_* can be measured directly. The local damping *Q*_loc_^−1^ is given by the ratio of the imaginary part of the contact stiffness to the real part. The above set of equations was solved by using the LabVIEW^®^ program, in order to obtain the real and imaginary parts of the wave vectors and the contact stiffness using the procedures employed by Yuya et al. [8,25] and Killgore et al. [11]. Here, it is convenient to introduce:

[3]
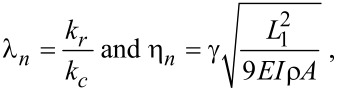


where λ*_n_* is the normalized contact stiffness and η*_n_* is the normalized damping constant. The parameter γ stands for the interaction damping in the contact zone, generally represented by a dashpot parallel to a spring. *I* is the area moment of inertia.

By using an appropriate contact mechanics model, one can convert the obtained stiffness values to the reduced elastic modulus E^*^ and then to the indentation modulus *M*. The contact mechanics for AFM tips is very difficult to model as the exact shape of the tip in contact with the sample is usually unknown. The Hertz model is a simplified and the most widely used contact mechanics model for AFM contacts. It assumes each contact to be an elastic half space with relatively small strains at the frictionless contact with elliptical shape [26]. The tip radius and the applied load are considered to be constant during a scan. Hence, the modulus of one phase could be considered as reference for obtaining the modulus of the other phases. If we assume the tip to be spherical, the real part of the contact stiffness, *k_r_*, is related to E^*^ through the following equation [1,8,12–13]:

[4]
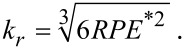


Here, E^*^ is the reduced elastic modulus, *R* is the radius of curvature of the cantilever tip and *P* is the load applied on the specimen through the cantilever. There is one unknown, *R*, which can be eliminated by using a reference method. Reference material can either be a single crystal with known orientations or an amorphous material. Recently, Phani et al. [12] have demonstrated an approach that circumvents the problem of the change in the tip condition by simultaneous acquisition of two contact-resonance frequencies and by selecting a few points in the matrix in each scan line as a reference. This methodology eliminates repeated switching between an unknown specimen and a reference specimen for quantitative measurement of the indentation modulus. The calibration procedure is based on the equation:

[5]
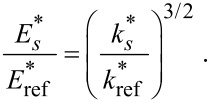



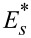
 is governed by the elastic properties of the specimen and the tip. The relation between the indentation moduli *M*_tip_ and *M*_sample_ and 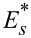 is given by:

[6]
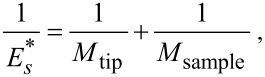


with

[7]
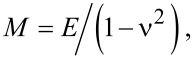


where ν is the Poisson’s ratio. By using the values obtained for *k_r_* and *k_i_* and, hence, λ*_n_* and η*_n_* from the dispersion equation, one can get the local damping *Q*_loc_^−1^ = *E*″/*E*′ values of the specimen at the nanometer scale. The damping for the first mode can be derived from the equation:

[8]
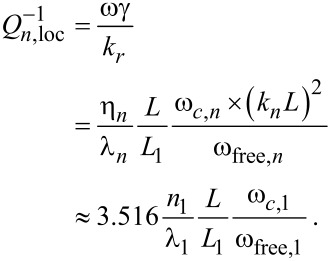


Here, ω and ω_free_ are the contact and the free angular resonance frequencies, respectively. The lower approximation of Equation 8 holds for the first cantilever mode.

## Experimental

Three specimens of Ti-6Al-4V of dimensions of about 25 × 25 × 4 mm^3^ were solution-annealed at 1323 K (above the β-transus temperature) for one hour, followed by water quenching. The solution-annealed specimens were then heat-treated at 923, 1123 and 1223 K, respectively, for one hour, followed by water quenching. The specimens were prepared by using an automatic polishing machine down to 0.25 µm diamond solution. Further, the specimens were polished by using a 50 nm colloidal silica suspension at a very low load to obtain the strain-free specimen surfaces. Plane parallelism of the specimens was maintained throughout the polishing.

An NTEGRA AFAM system supplied by M/s. NT-MDT Co., Zelenograd, Russia was used in the study. A stiff cantilever with a spring constant, *k*_c_, of about 30 N/m and the first free resonance frequency *f*_0_ of about 171 kHz was used in the study. The surface topography of the specimens was obtained in tapping mode to select an area with sufficient flatness for acquiring the contact-resonance spectra. A topographical variation is always observed for different phases upon mechanical/chemical-mechanical polishing of a sample due to difference in their mechanical/chemical properties. Even differences in the crystallographic orientation of the phase exhibit different topography upon polishing. In order to avoid excessive damage to the cantilever tip during contact scanning, an area with sufficient flatness was selected, where the maximum height variation for different phases was found to be less than 10 nm. An area of about 5 × 5 µm^2^ was selected for the AFAM measurements. The AFAM data was acquired by using cantilevers with similar dimensions and stiffness values. A step of 50 nm was chosen and the contact resonance spectrum of the first flexural mode was acquired with a resolution of about 0.1 Hz in the range of 650–850 kHz for all the specimens heat-treated at different temperatures. In order to effectively obtain the contribution of local internal friction *Q*_loc_^−1^ from the sample, the load of the cantilever onto the sample was chosen to be about 1200 nN for all the measurements. Caron et al. [2] have observed that a background damping in the material related to the global ultrasonic absorption is obtained at higher loads only. Hence, a slightly higher value of load was selected at which no noticeable wear of the tip and no slipping in the tip–sample contact were observed and, hence, uniform measurements throughout the scan were assured. The contact-resonance spectra were analyzed using software specifically developed in LabVIEW to obtain the maps of the indentation modulus and damping (i.e., local internal friction). The indentation modulus of the individual phases obtained by AFAM measurements was used to estimate the average modulus of the specimens, using the volume fractions of the phases present in the specimens. The volume fractions of the phases were estimated using JMatPro^®^ [27–28] simulation software. The volume fractions obtained by JMatPro^®^ were validated on one of the samples experimentally by X-ray diffraction (XRD) measurements, using an INEL make XRG3000 diffractometer operated at 40 kV and 30 mA with monochromatic Cu Kα_1_ radiation. The XRD spectrum was recorded in the 2θ range of 10 to 100° using a curved position-sensitive (CPS) detector with a step size of 0.011°.

In order to identify various phases present in the specimen microstructure, an electron back-scatter diffraction (EBSD) study was performed using a Zeiss SUPRA 55 Gemini field emission gun (FEG) scanning electron microscope (SEM) at an accelerating voltage of 20 kV, an aperture of 120 μm, a working distance of 16 mm, a tilt angle of 70° and a specimen–detector distance of 178 mm. An indexing algorithm based on eight detected bands was utilized.

## Results and Discussion

**Figure 2:**
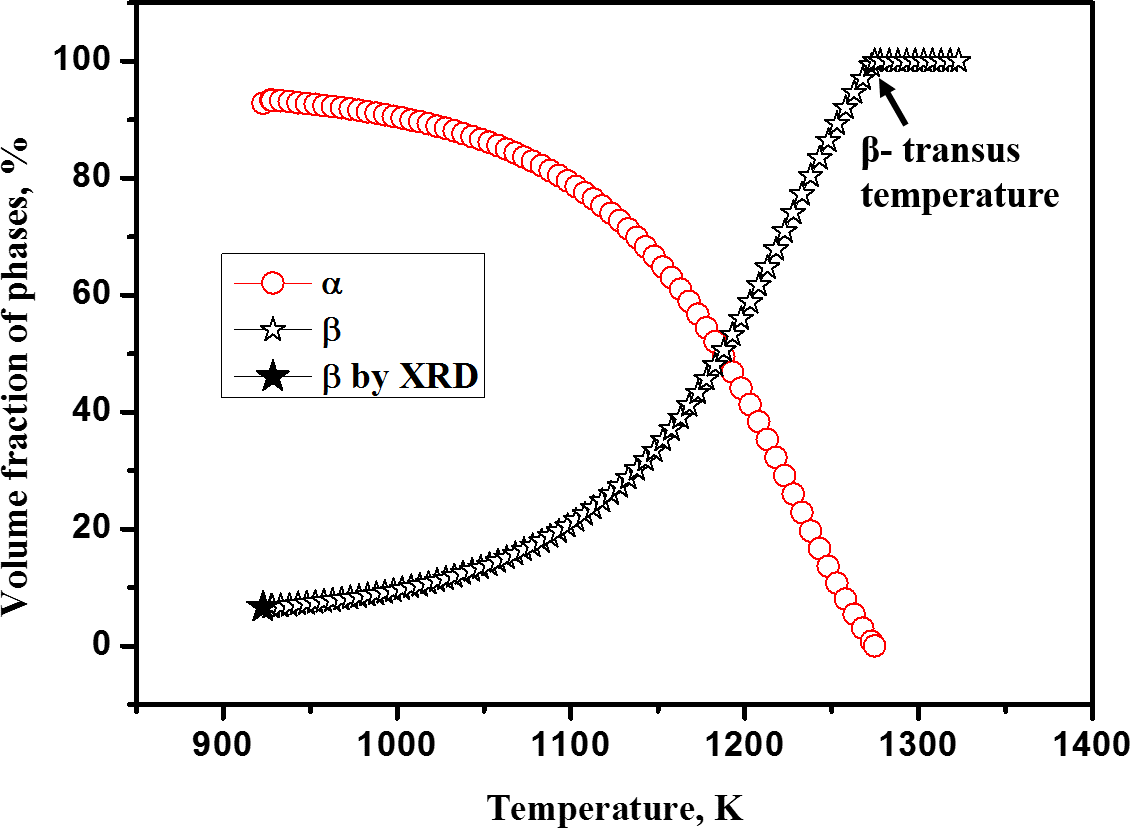
Variations in the volume fraction of α- and β-phases with the heat treatment temperature as obtained by the JMatPro^®^ simulation software and XRD measurement.

The amount of the α- and β-phases present at different heat-treatment temperatures for Ti-6Al-4V alloy, as obtained by the JMatPro^®^ simulation, is shown in Figure 2. The volume fraction of the β-phase is very low (ca. 6.57%) at 923 K and it increases to 26% and 70.9% at 1123 K and 1223 K, respectively. The JMatPro^®^ simulation indicates a β-transus temperature of 1275 K, above which only the β-phase is observed. This is in good agreement with the earlier reported experimental value of the β-transus temperature of 1268 K for the alloy [29].

**Figure 3:**
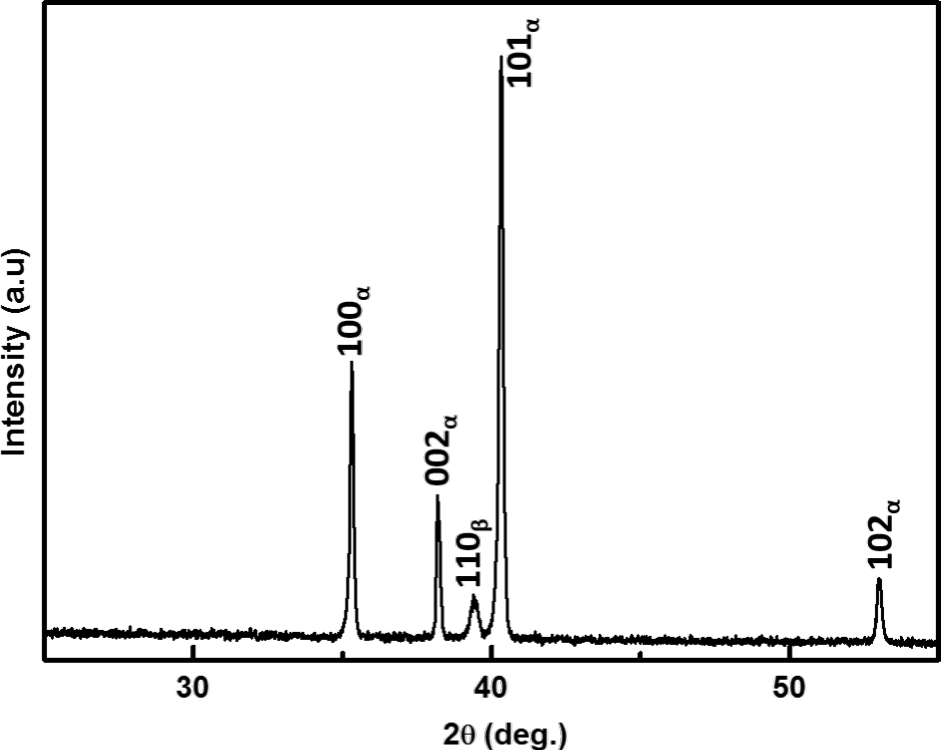
XRD spectrum obtained using the profile fitting method for Ti-6Al-4V sample heat-treated at 923 K for one hour, followed by water quenching.

Figure 3 shows the XRD spectrum for the specimen heat-treated at 923 K. The volume fraction of the β-phase obtained by using XRD is 6.61%, which is in excellent agreement with that obtained using JMatPro^®^.

**Figure 4:**
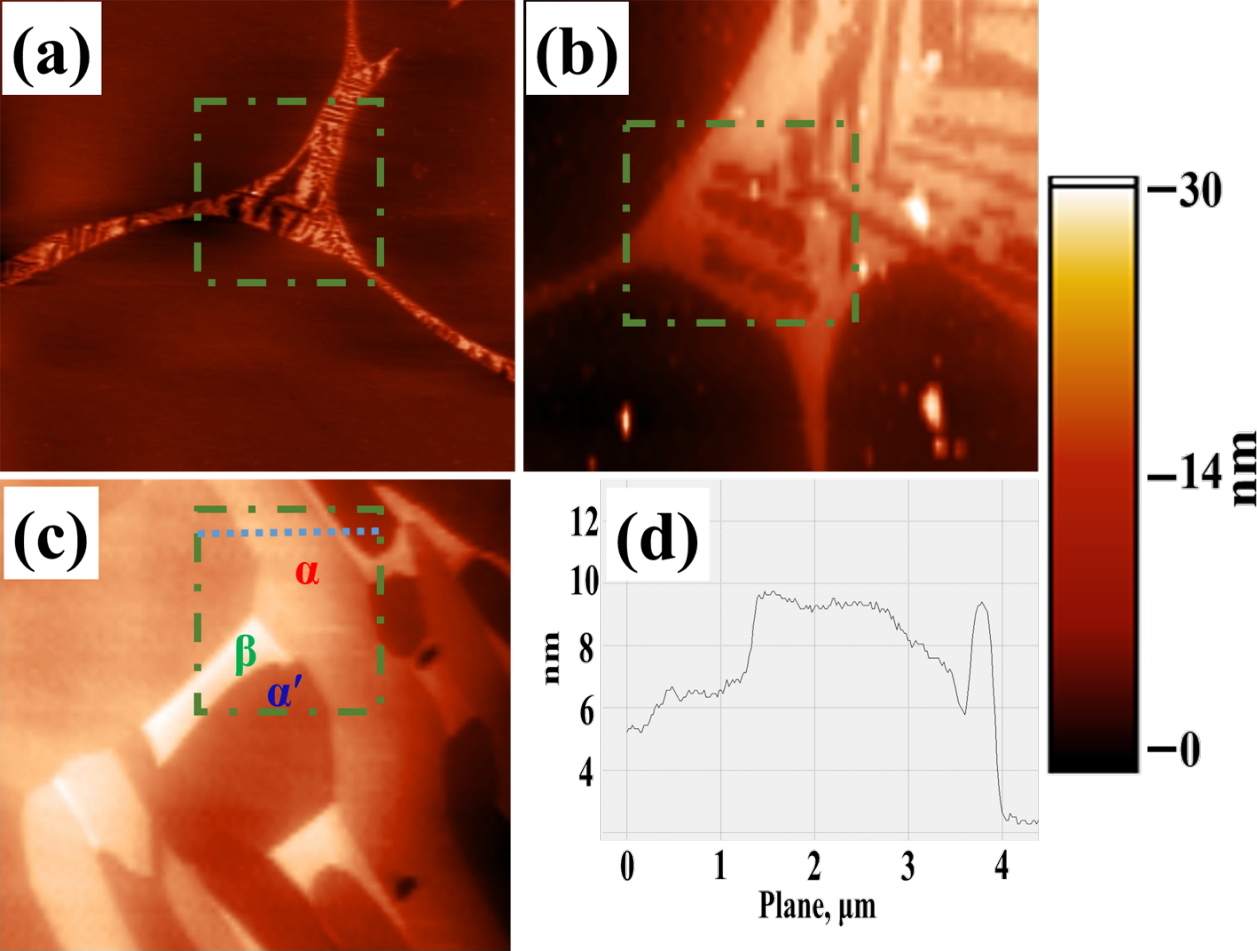
Topography of Ti-6Al-4V specimens heat-treated at (a) 923 K; (b) 1123 K; (c) 1223 K for one hour followed by water quenching and (d) topography line-profile corresponding to the dotted line marked in (c).

Typical AFM topography images for the specimens heat-treated at 923, 1123 and 1223 K are shown in Figure 4a–c, respectively. The topography indicates the presence of two different phases in the specimens heat-treated at 923 and 1123 K. The increase in the size and volume fraction of the bright phase (with higher topography) can be clearly seen in the topography image of the specimen heat-treated (SHT) at 1123 K (Figure 4b), as compared to that for the SHT at 923 K (Figure 4a). Based on the volume fraction of phases, the brighter phase is identified as the β-phase. Based on the topography, three different phases can be observed in the SHT at 1223 K (Figure 4c). The topography line profile corresponding to the dotted line (in blue) shown in Figure 4c is given in Figure 4d. A maximum of 10 nm height variation is observed in Figure 4d for the different phases. The surface roughness (*S*_RMS_) is found to be less than 0.33 nm for the individual phases.

**Figure 5:**
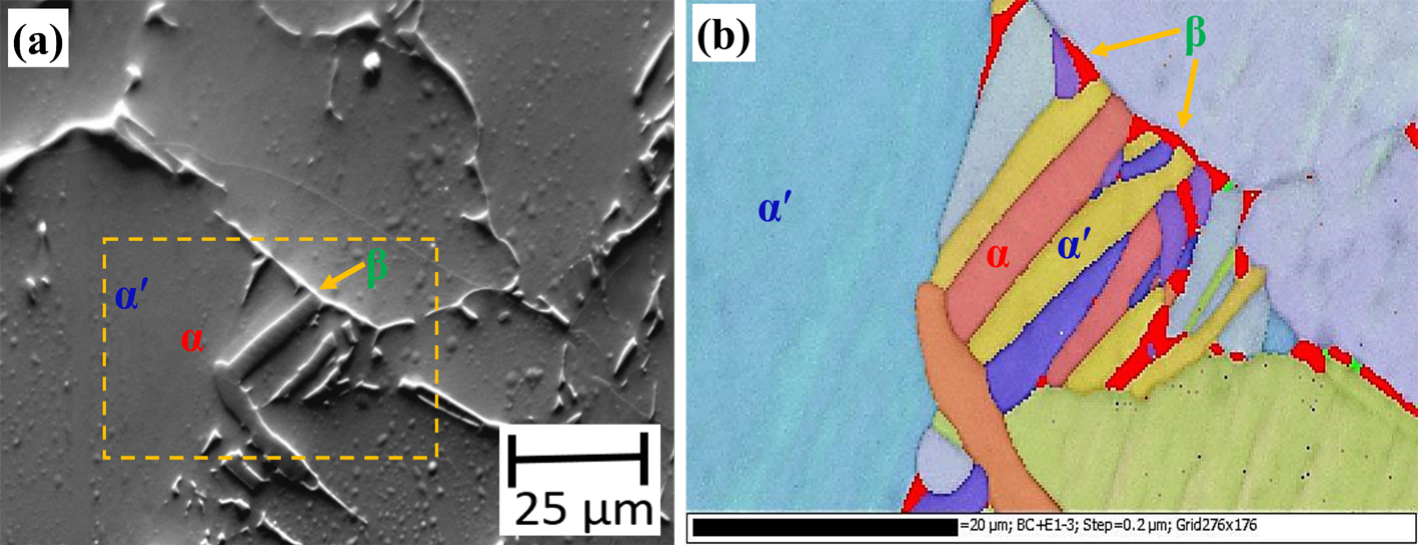
(a) Topography image and (b) a composite image showing typical microstructure in a Ti-6Al-4V specimen heat-treated at 1223 K for one hour followed by water quenching. The topography image is obtained by a forward scattering detector in a FEG-SEM. The composite image consists of an inverse-pole-figure map along with band contrast for the HCP (α and α′) phases and the BCC (β) phase is shown in red.

In order to unambiguously identify these phases, SEM and EBSD studies were carried out on this specimen. Figure 5a shows the topography image obtained by using a forward scattering detector (FSD) in the FEG-SEM. The topography image indicates the presence of three different phases viz., the matrix with the low topography, a lath-like phase with intermediate height and a bright phase at the boundary. The EBSD analysis indicates that both the matrix and the lath-like phase with intermediate height are of hexagonally closed package (HCP) structure, whereas, the phase at the boundary is of body-centered cubic (BCC) structure. This can clearly be seen in the composite image shown in Figure 5b, which consists of an inverse-pole-figure map along with the band contrast for the HCP phases and the BCC phase is shown in red. The matrix is identified as α′ martensite with HCP structure, which the high temperature β-phase in an α + β titanium alloy transforms to, upon quenching from temperature above about 1123 K [29]. The presence of untransformed β-phase along with α′ matrix and α lath has also been reported by Pederson [30] in Ti-6Al-4V alloy heat-treated at 1223 K and followed by water quenching. With the help of the EBSD analysis, it becomes clear that the matrix in Figure 4c is α′ martensite and the two phases with intermediate and high topography are the α- and β-phases, respectively.

**Figure 6:**
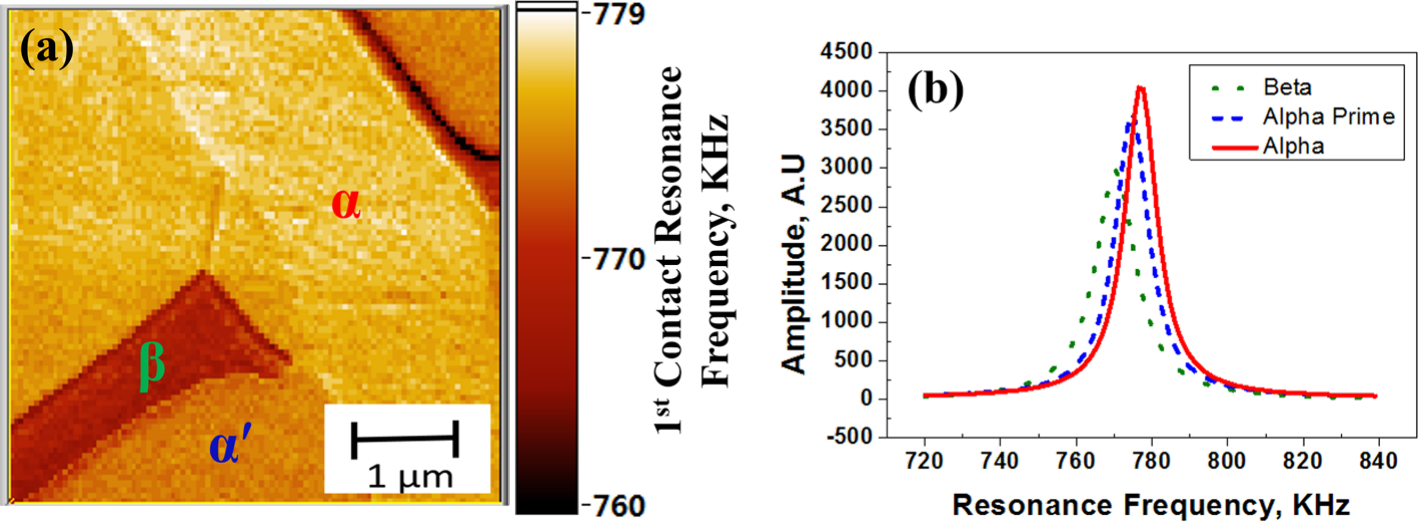
(a) First contact resonance frequency map for a Ti-6Al-4V specimen heat-treated at 1223 K for one hour, followed by water quenching and (b) the typical resonance spectra for the three different phases identified as α, β, and α′.

A map of the peak frequency in the contact-resonance spectra for the SHT at 1223 K is shown in Figure 6a. The presence of three phases with different contact-resonance frequencies can be clearly seen in Figure 6a. Figure 6b shows the typical contact-resonance spectra for the three phases. The α′-matrix phase exhibited an intermediate value of the contact-resonance frequency, whereas, the α lath and the retained β exhibit the highest and the lowest contact-resonance frequencies, respectively. This indicates that the α-phase has the highest modulus followed by the α′- and β-phases, respectively.

This is in agreement with the values reported on the basis of ultrasonic velocity measurements on bulk samples heat-treated at different temperatures in the range of 923 to 1323 K [31]. Table 1 and Table 2 summarize the elastic properties and ultrasonic attenuation values obtained in the bulk specimens heat-treated at 923, 1123, 1223 and 1323 K. The modulus was found to be the highest in the sample heat-treated at 923 K that has the maximum volume fraction of the α-phase. The attenuation was found to be highest in the sample heat-treated at 1223 K that has the maximum amount of β-phase. The SHT at 1323 K is comprised of a single-phase α′ microstructure. Hence, the isotropic indentation modulus of the α′-phase can be obtained by using the bulk *E* and ν values of the SHT at 1323 K. Kumar et al. [13] reported that the isotropic indentation modulus obtained with the bulk measurements does not vary much with the anisotropic modulus measured on different crystallographic planes. By using a similar approach, the isotropic indentation modulus of the α′-phase is used as a reference for obtaining the indentation modulus of the other phases in the present study. The *M* value for the α′-phase as determined by the ultrasonic velocity measurements is 127.8 GPa [31].

**Table 1:** Ultrasonic velocity measurements obtained from literature for Ti-6Al-4V bulk samples heat-treated at different temperatures.

temperature [K]	volume fraction (%) by JMatPro^®^ simulation			ultrasonic data on bulk sample [23]			

	α	β	α′	*E* [GPa]	attenuation [dB/mm]	Poisson’s ratio ν	*M*_BS_ [GPa]

923	93.3	6.6	0	115.7	0.36	0.321	132.7
1123	74	26	0	111.5	0.42	0.326	130
1223	29	5^a^	66^a^	114	0.36	0.323	128.8
1323	0	0	100	114.5	0.38	0.323	127.8 (ref)

^a^The volume fraction of the retained β-phase given is approximate, based on the AFM and SEM microstructure. The volume fraction of the α′-phase is calculated based on the difference in the volume fraction of the β-phase estimated by the JMatPro^®^ and the volume fraction of the retained β-phase. The values of the Poisson’s ratio are ν_α_ = 0.32 and ν_β_ = 0.33.

**Table 2:** AFAM measurements obtained for the individual phases present in Ti-6Al-4V samples heat-treated at different temperatures.

temperature [K]	AFAM measurements						

	*M*_α_ [GPa]	*M*_β_ [GPa]	*E*_α_ [GPa]	*E*_β_ [GPa]	*E*_BS_ [GPa]	(*E′′*/*E′*)_α_	(*E′′*/*E′*)_β_

923	133.5	120	119.7	106.9	119.1	0.013	0.028
1123	133.5	118.7	119.7	105.7	116.2	0.044	0.056
1223	133.5	117	119.7	104.2	115.4	0.019	0.028

**Figure 7:**
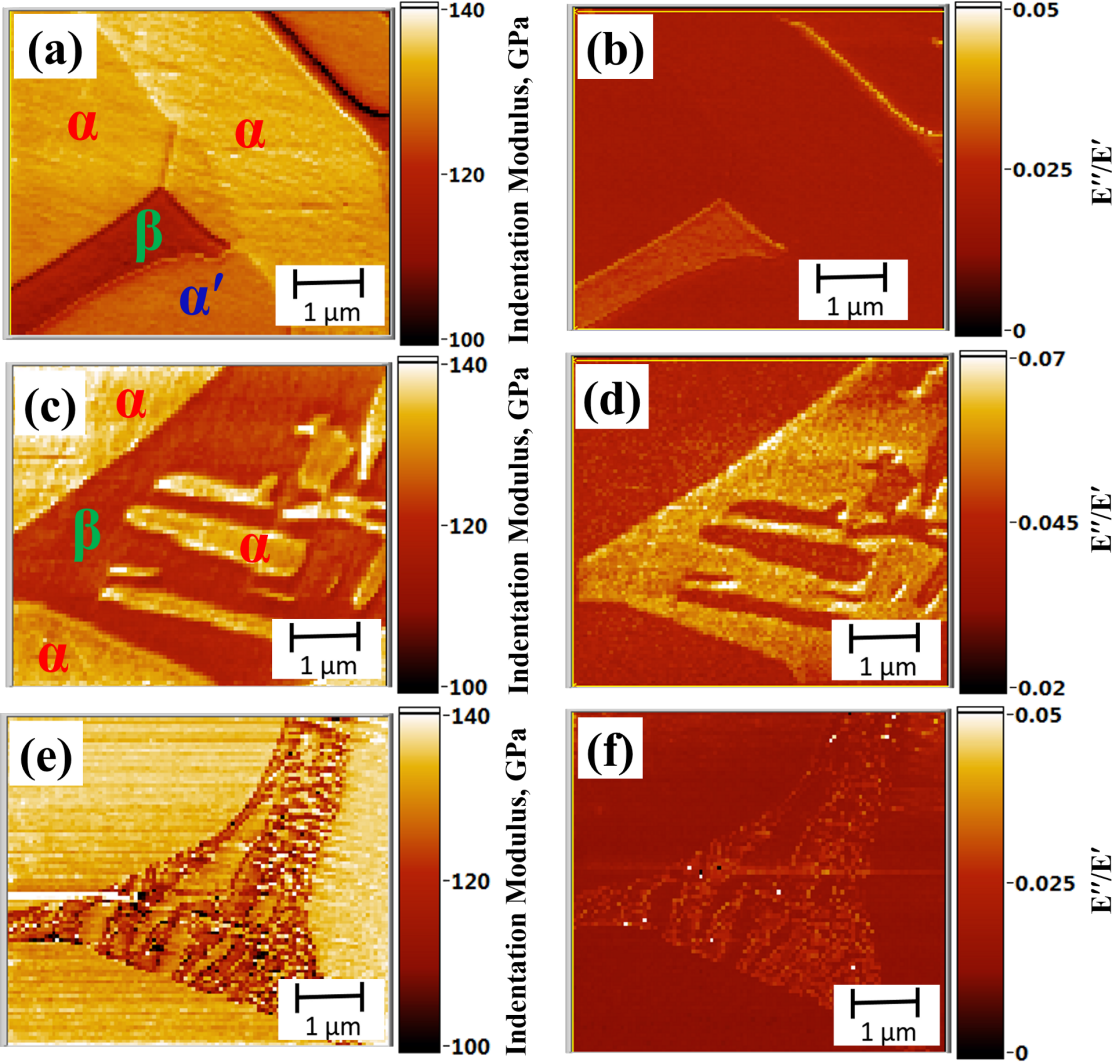
Modulus and damping maps of the Ti-6Al-4V specimen’s heat-treated at (a and b) 1223 K, (c and d) 1123 K, and (e and f) 923 K for 1 h, followed by water quenching, respectively.

By using the software developed in LabVIEW^®^ incorporating the cantilever dynamics model (Equations 1–3), the contact-stiffness map is obtained based on the contact-resonance frequencies shown in Figure 6a. After obtaining the *k_r_* map, a small area in the *α*′ region was selected as a reference to obtain the indentation modulus map of the SHT at 1223 K, as shown in Figure 7a. Statistical analysis was carried out on the data of Figure 7a to obtain the average (mode) values of *M* for α- (*M*_α_) and β- (*M*_β_) phases in the SHT at 1223 K, which are found to be 133.5 GPa and 117 GPa, respectively. The three phases are labeled in the figure for better understanding. The modulus of the α-phase is reported to be less influenced by the compositional variations [31], hence the *M*_α_ obtained for the 1223 K sample is used as a reference to get the average *M*_β_ in the other two SHT at 1123 K and 923 K.

Figure 7c shows the modulus map for the SHT at 1123 K. Using the *M*_α_ obtained for the SHT at 1223 K as a reference, the average value for *M*_β_ was obtained as 118.7 GPa. Figure 7e shows the modulus map for the SHT at 923 K. The average value for *M*_β_ is obtained as 120 GPa. The relative modulus values measured using AFAM is found to be similar for α- and β-phases in the specimens heat-treated at different temperatures. The Young’s modulus of the individual phases can be approximated by using *M* values for the respective phases and Equation 6. Poisson’s ratio values as a function of heat-treatment temperature for Ti-6Al-4V samples are discussed in detail elsewhere [31]. The Poisson’s ratios used for the calculations for the α and β phases are ν_α_ = 0.32 and ν_β_ = 0.33, respectively. By knowing the *E* and volume fraction of each phase, the average *E* of the bulk sample can be calculated using the rule of mixture as given below [32]:

[9]
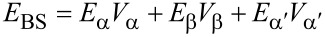


where *V*_α_, *V*_β_ and *V*_α′_ are the volume fractions of α, β and α′ phases in the sample and *E*_BS_ is the average Young’s modulus of the bulk sample.

Substituting the values for *E*_α_ and *E*_β_*,* obtained by AFAM and *V*_α_ and *V*_β_ obtained by JMatPro^®^ simulation for specimens heat-treated at 923, 1123 and 1223 K in Equation 9, the *E*_BS_ values are obtained as 119.1, 116.2 and 115.4 GPa, respectively. These values are compared with those obtained with the bulk ultrasonic velocity measurements averaging over the whole sample, in Table 1. The maximum deviation in the *E*_BS_ values calculated using the AFAM is 5% compared to those calculated by using ultrasonic velocity measurements. This variation is essentially attributed to the effect of crystallographic orientation on the modulus, which is not considered in the present study.

In Figure 6b, the width of the contact-resonance spectrum is larger by about 30% for the β-phase than for the α′- and α-phases, indicating a higher damping in the β-phase for the sample heat-treated at 1223 K. Therefore, along with the modulus, damping was also mapped simultaneously for all three heat-treated specimens. Equations 1–3 and Equation 8 have been used for calculation of the damping in different samples.

Figure 7 (panels b, d and f) shows the damping maps for specimens heat-treated at 923, 1123 and 1223 K. For all the three heat-treated samples, the β-phase exhibits the highest damping followed by α′ and α. The obtained results for damping are in line with the ultrasonic attenuation measurements carried out on bulk Ti-6Al-4V samples heat-treated at 923, 1123 and 1223 K, as shown in Table 1 [31]. The SHT at 1123 K having the highest amount of β-phase exhibited the maximum attenuation. Even though the absolute values of the damping are not the same for the phases in the three specimens, the relative damping values for of the α- and the β-phase are similar in all three samples. The higher absolute value of damping in the SHT at 1123 K is attributed to the larger tip radius, as is evident from the contact-resonance frequency values. The first contact-resonance frequency values for the SHT at 1123 K was found to be in the range of 821–837 kHz, as compared to 710–750 kHz for the SHT at 923 K and 760–780 kHz for the SHT at 1223 K. The contact-radius values calculated by using Equation 4 for the measurements made on the SHT at 923, 1123 and 1223 K are found to be 27.4, 28.1 and 63.8 nm, respectively. The study demonstrates the uniqueness of AFAM for modulus and damping mapping in multiphase structural alloys with a lateral spatial resolution of better than 50 nm.

## Conclusion

Simultaneous mapping of elastic and damping properties in a multiphase structural alloy by using an AFM technique is reported for the first time in the present study, in which a cantilever dynamics model is used that also considers damping. The β-phase exhibited minimum modulus and maximum damping followed by α′- and α-phases in all three heat-treated Ti-6Al-4V alloy samples. The relative modulus values measured by using AFAM are found to be similar for α- and β-phases in the specimens heat-treated at different temperatures. The study also demonstrates that the micro-scale elastic properties measured by using AFAM can also be used for obtaining the average elastic properties of the bulk samples.
